# The genome sequence of sweet cherry (*Prunus avium*) for use in genomics-assisted breeding

**DOI:** 10.1093/dnares/dsx020

**Published:** 2017-05-25

**Authors:** Kenta Shirasawa, Kanji Isuzugawa, Mitsunobu Ikenaga, Yutaro Saito, Toshiya Yamamoto, Hideki Hirakawa, Sachiko Isobe

**Affiliations:** 1 Kazusa DNA Research Institute, Kisarazu, Chiba 292-0818, Japan; 2 Horticultural Experiment Station, Yamagata Integrated Agricultural Research Center, Sagae, Yamagata 991-0043, Japan; 3 Central Agricultural Experiment Station, Agricultural Research Department, Hokkaido Research Organization, Naganuma, Hokkaido 069-1395, Japan; 4 Institute of Fruit Tree and Tea Science, National Agriculture and Food Research Organization, Tsukuba, Ibaraki 305-8605, Japan

**Keywords:** draft genome, genetic map, genomics-assisted breeding, sweet cherry (*Prunus avium*)

## Abstract

We determined the genome sequence of sweet cherry (*Prunus avium*) using next-generation sequencing technology. The total length of the assembled sequences was 272.4 Mb, consisting of 10,148 scaffold sequences with an N50 length of 219.6 kb. The sequences covered 77.8% of the 352.9 Mb sweet cherry genome, as estimated by *k*-mer analysis, and included >96.0% of the core eukaryotic genes. We predicted 43,349 complete and partial protein-encoding genes. A high-density consensus map with 2,382 loci was constructed using double-digest restriction site–associated DNA sequencing. Comparing the genetic maps of sweet cherry and peach revealed high synteny between the two genomes; thus the scaffolds were integrated into pseudomolecules using map- and synteny-based strategies. Whole-genome resequencing of six modern cultivars found 1,016,866 SNPs and 162,402 insertions/deletions, out of which 0.7% were deleterious. The sequence variants, as well as simple sequence repeats, can be used as DNA markers. The genomic information helps us to identify agronomically important genes and will accelerate genetic studies and breeding programs for sweet cherries. Further information on the genomic sequences and DNA markers is available in DBcherry (http://cherry.kazusa.or.jp (8 May 2017, date last accessed)).

## 1. Introduction

Sweet cherry (*Prunus avium*, 2n = 2x = 16) and its tetraploid relatives (*Pr. cerasus* and *Pr. pseudocerasus*, 2n = 4x = 32) are fruit crops of the Rosaceae family, which also includes apple (*Malus *×* domestica*), peach (*Pr. persica*), Japanese apricot (*Pr. mume*), strawberry (*Fragaria vesca* and *F. *× *ananassa*), and Japanese, Chinese, and European pears (*Pyrus pyrifolia*, *Py. bretschneideri*, and *Py. communis*). Because of their economic importance, e.g. the world production of 2.2 M tonnes in 2014 (FAOSTAT: http://www.fao.org/faostat (8 May 2017, date last accessed)), breeding programs for fruit crops are progressing all over the world. However, in general, their breeding efficiency has lagged behind that of the cereal crops and vegetables due to the time and space required to grow them. Because genomics-based breeding could overcome this drawback, whole-genome sequencing has been performed on a number of the Rosaceae crops, including apple,^1^ peach,^2^ pear,^3^^,^^4^ Japanese apricot,^5^ and strawberry,^6^^,^^7^ as well as on >100 other plant species.^8^

Nevertheless, whole-genome sequencing of sweet cherry has not been reported despite its simple, compact genome (2n = 2x = 16, genome size of ∼380 Mb). According to sweet cherry genetic maps, the structure of the sweet cherry genome is predicted to be similar to that of the peach genome,^9^ meaning that the order of markers is conserved between the two species. Moreover, the positions of QTLs for agronomically important traits (e.g. disease resistances, as well as flower, vegetative, and fruit or nut quality) overlap.^10^ Therefore, genomic information from peaches, as well as other Rosaceae fruiting crops, has already been utilized in sweet cherry breeding.^11^

Even though the marker orders are conserved between sweet cherries and peaches, the genome sequences have diverged.^12^ A conserved set of orthologous markers bridge the barrier between the genome sequences, but the number of available markers is limited,^13^ forcing researchers and breeders to develop a high-throughput SNP genotyping system.^14^ To enhance the breeding programs for sweet cherries and to assist future genetics and genomics studies, we established genomic resources such as whole-genome sequence data, a high-density genetic map, the sweet cherry pseudomolecule based on the genetic map and synteny with the peach genome, and DNA markers using SNPs, simple sequence repeats (SSRs), and insertions/deletions identified from whole-genome resequencing of six modern cultivars. In addition, we identified agronomically important genes for fruit color, morphology, and quality and self-incompatibility in the genome. This study will be useful for breeding programs and in genetics and genomics studies on not only sweet cherry but also other members of the Rosaceae family including the sweet cherry relatives, *Pr. cerasus* and *Pr. pseudocerasus*, both of which possess complex genomes due to tetraploidy. 

## 2. Materials and methods

### 2.1. Sequencing analysis of the sweet cherry genome

A Japanese leading variety of sweet cherry (*Pr. avium*) (i.e. Satonishiki) was used for genomic sequencing. For genomic diversity analysis, six Japanese varieties (Benikirari, Benisayaka, Benishuho, Benitemari, Beniyutaka, and Nanyo) were used. The pedigree of the materials is shown in Supplementary Figure S1. Young leaves from each variety were collected from the original trees (Benikirari, Benitemari, and Beniyutaka) and clones (Satonishiki, Benisayaka, Benishuho, and Nanyo), all of which were planted in the Horticultural Experiment Station at Yamagata Integrated Agricultural Research Center, Japan.

Genomic DNA was extracted from the leaves using a DNeasy Plant Mini Kit (Qiagen, Hilden, Germany) and used for construction of a paired-end (PE) library (insert size: 500 bp), in accordance with the TruSeq DNA Sample Preparation Guide (Illumina, San Diego, CA, USA). In addition, four mate-pair (MP) libraries (insert sizes of 2, 5, 10, and 15 kb) were constructed with GS Titanium Library Paired End Adaptors (Roche, Basel, Switzerland).^15^ The nucleotide sequences were determined using massively parallel sequencing by synthesis on a HiSeq2000 (Illumina) in PE 93 bp mode.

### 2.2. Genome size estimation and genome assembly

Out of the obtained sequence reads for PE and MP sequencing, low-quality reads were removed and adapter sequences were trimmed using PRINSEQ^16^ (version 0.20.4: parameters of -trim_right 1, -trim_qual_right 10, and -min_len 92) and fastx_clipper (parameter of -a AGATCGGAAGAGC) in the FASTX-Toolkit (version 0.0.14: http://hannonlab.cshl.edu/fastx_toolkit (8 May 2017, date last accessed)), respectively. The filtered high-quality reads were used for genomic size estimation based on *k*-mer frequency (*k* = 17) using Jellyfish^17^ (version 2.1.4).

The high-quality PE reads were assembled into contigs using SOAPdenovo2^18^ (version r240: parameters of -F and -R) or Platanus^19^ (version 1.2.1). In the assembly with SOAPdenovo2, *k*-mer sizes from 51 to 91 were examined. After comparing the two assemblies, sequence data obtained from SOAPdenovo2 (*k*-mer = 81) were chosen for scaffolding with high-quality MP reads, which was carried out using SOAPdenovo2. Gaps, represented by Ns in the sequence, were filled with the high-quality PE reads using GapCloser^18^ (version 1.10: parameter of -p 31). Contaminating sequences were removed by searching with BLASTN, with an E-value cutoff of 1E − 10 and length coverage of ≥10%, against sequences from potential contaminating sources such as organelles (chloroplasts of peach [accession number: HQ336405], Japanese apricot [accession number: KF765450], and strawberry [accession number: NC_015206], and mitochondria of *Arabidopsis* [accession number: NC_001284] and apple [accession number: NC_018554]), other organisms (bacterial and fungi genome sequences registered in NCBI [http://www.ncbi.nlm.nih.gov], and the human genome [hg19]), and artifacts (Illumina PhiX Sequencing Control v3 and vector sequences from UniVec [http://www.ncbi.nlm.nih.gov/tools/vecscreen/univec/ (8 May 2017, date last accessed)]). The resulting sequences that were ≥1,000 bases were selected and designated PAV_r1.0. Completeness of the assembly was assessed with sets of Benchmarking Universal Single-Copy Orthologues (BUSCO)^20^ (version 1.1b).

### 2.3. Repetitive sequence analysis

Repetitive sequences in PAV_r1.0 were identified using Repbase.^21^ A *de novo* repeat library for PAV_r1.0 was built using RepeatScout^22^ (version 1.0.5), and the repetitive sequences were searched for using RepeatMasker^23^ (version 4.0.3) based on known repetitive sequences registered in Repbase^21^ and the *de novo* repeat libraries.

### 2.4. RNA sequencing and assembly

Total RNA was extracted from leaves, roots, flowers, calli, brown rot fruits infected by *Monilinia fructicola*, and fruits from three different stages (32 days after full bloom [DAFB], yellow; 44 DAFB, initial red; and 54 DAFB, full red)^24^ using the RNeasy Mini Kit (Qiagen) or phenol/SDS extraction, and then treated with RQ1 RNase-Free DNase (Promega, Madison, WI, USA) to remove contaminating genomic DNA. RNA libraries were constructed in accordance with the TruSeq Stranded mRNA Sample Preparation Guide (Illumina). The nucleotide sequences were determined using massively parallel sequencing by synthesis on a MiSeq (Illumina) in the PE 301 bp mode. The obtained reads were treated as above to remove low-quality reads and to trim adapter sequences, and were assembled using Trinity^25^ (version r20140717: parameters of –min_contig_length 100, –group_pairs_distance 400, and –SS_lib_type RF) to generate a UniGene set.

### 2.5. Gene prediction and annotation

Transfer RNA (tRNA) genes were predicted using tRNAscan-SE^26^ (version 1.23) with the default parameters, whereas ribosomal RNA (rRNA) genes were predicted using BLASTN searches with an E-value cutoff of 1E − 10, with the *Arabidopsis thaliana* 18S rRNA (accession number: X16077) and 5.8S and 25S rRNAs (accession number: X52320) used as query sequences.

To identify putative protein-encoding genes in PAV_r1.0, a MAKER pipeline^27^ (version 2.31.8) including *ab-initio*-, evidence-, and homology-based gene prediction methods was used. For this prediction, the UniGene set generated from the RNA-Seq analysis and peptide sequences predicted from the genomes of Rosaceae members (e.g. *F. vesca* [Genome Database for Rosaceae, GDR, version v2.0.a1],^28^*Pr. persica* [GDR v2.0.a1]^2^, *M.* × *domestica* [GDR v1.0p],^1^ and *Pr. mume*^5^) were used as a training data set. In addition, BRAKER1^29^ (version 1.3) was also used to complete the gene set for PAV_r1.0. Genes related to transposable elements (TEs) were detected using BLASTP searches against the NCBI non-redundant (nr) protein database with an E-value cutoff of 1E − 10 and by using InterProScan^30^ (version 4.8) searches against the InterPro database^31^ with an E-value cutoff of 1.0. The putative genes of PAV_r1.0 were clustered using CD-hit^32^ (version 4.6.1) with the UniGene set of *F. vesca* (GDR v2.0.a1),^28^*Pr. persica* (GDR v2.0.a1)^2^, *M.* × *domestica* (GDR v1.0p),^1^ and *Pr. mume*^5^ with the parameters c = 0.6 and aL = 0.4. The genes in the plant species described above were classified into plant gene ontology (GO) slim categories^33^ and euKaryotic clusters of Orthologous Groups (KOG) categories,^34^ and mapped onto the Kyoto Encyclopedia of Genes and Genomes (KEGG) reference pathways.^35^

### 2.6. Construction of genetic linkage maps and comparative genomics

Three F1-mapping populations, shown in Supplementary Figure S1, were used to construct genetic linkage maps: (1) C-303 (*n* = 94), derived from a cross between Beniyutaka and Benikirari; (2) C-309 (*n* = 84), derived from a cross between C-195-50, which is a hybrid of Benishuho and an F1 C-47-70 of Benisayaka × Rainer, and Benikirari; and (3) HRO (*n* = 384), derived from a cross between Nanyo and Benisayaka. Genomic DNA extracted from the leaves of each line was subjected to double-digest restriction site–associated DNA sequencing (ddRAD-Seq) library construction.^36^ The DNA was digested using two restriction enzymes, *PstI* and *EcoRI*, and DNA fragments of 300–900 bp in length were fractionated using BluePippin (Sage Science, Beverly, MA, USA). The libraries were sequenced on a HiSeq (Illumina) in PE 93 bp mode.

Primary data processing of the sequencing reads was performed as described by Shirasawa et al.^36^ with minor modifications. Low-quality sequences were removed and adapters were trimmed using PRINSEQ^16^ (version 0.20.4) and fastx_clipper in the FASTX-Toolkit (version 0.0.13: http://hannonlab.cshl.edu/fastx_toolkit (8 May 2017, date last accessed)), respectively. The filtered reads were mapped onto the PAV_r1.0 reference sequence using Bowtie 2^37^ (version 2.2.3). To obtain a variant call format (VCF) file including SNP information, the sequence alignment/map format (SAM) files were converted to binary sequence alignment/map format (BAM) files and subjected to SNP calling using the mpileup command of SAMtools^38^ (version 0.1.19) and the view command of BCFtools.^38^ Missing data were imputed using Beagle4^39^ (version r1185). High-confidence SNPs were selected using VCFtools^40^ (version 0.1.12b) with the following criteria: 1) ≥5× coverage in each plant line (–minDP 5), 2) >10 SNP quality value (–minQ 10), 3) ≥0.2 minor allele frequency (–maf 0.2), and 4) <0.5 missing data rate (–max-missing 0.5).

In addition, SSR markers reported in the previous studies^41–56^ (Supplementary Table S1) were also employed. The polymorphism screening and genotyping were performed with an Applied Biosystems 3500 Series Genetic Analyzer (Applied BioSystems, Foster City, CA, USA). The segregated SNP and SSR data of the mapping population were prepared for the CP mode of JoinMap^57^ (version 4) and classified into groups using the Grouping Module of JoinMap with LOD scores of 4 to 7. The marker order and relative map distances were calculated using its regression-mapping algorithm with the following parameters: Haldane’s mapping function, ≤0.35 recombination frequency, and ≥2.0 LOD score. LPmerge^58^ (version 1.5) was used to integrate the linkage maps into the consensus map. The graphical linkage maps were drawn using MapChart^59^ (version 2.2).

For comparing the genome of sweet cherry with those of its relatives, similarity searches between the SNP-associated sequences of PAV_r1.0 (201 bp in length) and the pseudomolecule sequences of *Pr. persica* (GDR v2.0.a1),^2^*P. mume*,^5^*F. vesca* (GDR v2.0.a1),^28^ and *P. bretschneideri*^3^ were carried out using BLASTN searches with an E-value cutoff of 1E–15. The graphical comparative maps were drawn using Circos^60^ (version 0.69-3).

### 2.7. Pseudomolecule construction

Two approaches, based on the genetic map and synteny between the genomes of sweet cherry and peach, were used to construct pseudomolecule sequences. First, the genome scaffolds were assigned to the genetic map. If more than two marker loci were mapped on a single scaffold, the scaffolds were assigned with the orientation based on the marker order. Next, sequence similarity analysis of peptide sequences predicted from PAV_r1.0 was performed against those of the peach genome using BLASTP with an E-value cutoff of 1E–5. Scaffolds having a linear relationship (*R*^2^ > 0.6) with at least five continuous genes between the two genomes were assigned to a chromosome with that orientation. The resulting pseudomolecule sequences were aligned to the peach genome, GDR v2.0.a1, with NUCmer of the MUMmer package^61^ (version 2.23).

### 2.8. Whole-genome resequencing for identification of DNA polymorphism

Sequence reads from the PE sequencing of six varieties, Benikirari, Benisayaka, Benishuho, Benitemari, Beniyutaka, and Nanyo, were trimmed and filtered as above, and mapped on the PAV_r1.0 reference sequence with Bowtie 2^37^ (version 2.2.3: parameters of –minins 100, –no-mixed, and -k 2). The resulting BAM files were subjected to SNP calling with the mpileup command of SAMtools^38^ (version 0.1.19: parameter of -Duf) and the view command of BCFtools^38^ (parameter of -vcg), and filtered with VCFtools^40^ (version 0.1.12b: parameters of –minQ 50, –minGQ 20, –minDP 10, and –maxDP 100). The effects of mutations on gene function were predicted with SnpEff^62^ (version 4.2: parameters of -no-downstream and -no-upstream). SnpEff predicted the sequence ontology^63^ of the mutations and assigned them to four predefined impact categories: high- (e.g. nonsense mutations and frameshift mutations), moderate- (e.g. missense mutations), modifier- (e.g. intron and intergenic mutations) and low-impact (e.g. synonymous mutations) (see http://snpeff.sourceforge.net (8 May 2017, date last accessed) for details).

Copy number variations (CNVs) were detected with CNV-seq^64^ (version 0.2.7: parameter of –genome-size 272361615) using the BAM files, in which the six varieties were used as test lines with PAV_r1.0 as a reference.

### 2.9. Development of CAPS, indel, and SSR markers

SNP2CAPS^65^ was used for developing cleaved amplified polymorphic sequence (CAPS) markers with 19 restriction enzymes: *Afa*I, *Alu*I, *Apa*I, *Bam*HI, *Bgl*II, *Dra*I, *Eco*RI, *Eco*RV, *Hae*III, *Hha*I, *Hin*dIII, *Kpn*I, *Mbo*I, *Msp*I, *Pst*I, *Sac*I, *Sal*I, *Xba*I, and *Xho*I. SSRs were identified using the mismatched variable penalty (mmvp) mode of SciRoKoCo^66^ to detect imperfect microsatellites. Indels were selected from the VCF file of the resequencing analysis with VCFtools^40^ (version 0.1.12b: parameter of –keep-only-indels). Oligonucleotides for the markers were designed using PRIMER3^67^ (version 2.2.3).

## 3. Results

### 3.1. Sequencing and assembly of the sweet cherry genome

A total of 357.5 million high-quality reads (32.9 Gb) were obtained from the Satonishiki cherry PE library, which had an insert size of 500 bp (Supplementary Table S2). The distribution of distinct *k*-mers (*k* = 17) showed two peaks at multiplicities of 41 and 77 (Supplementary Fig. S2). The low and high peaks represent heterozygous and homozygous sequences, respectively, suggesting that the heterogeneity of the genome was low. We estimated the genome size to be 352.9 Mb from the higher peak, which almost agreed with the value measured by flow cytometry, 338 Mb.^68^

The 357.5 million PE reads were assembled into contigs using SOAPdenovo2 with five *k*-mer sizes (51, 61, 71, 81, and 91), and the obtained contigs were assembled into scaffolds with 121.3 million MP reads (Supplementary Table S2). When a *k*-mer size of 81 was employed, the total length of the scaffolds (373.7 Mb) was close to the estimated genome size and the N50 length (114.8 kb) was the longest. In parallel, we investigated another assembling tool, Platanus. However, while the N50 length (462.8 kb) was longer than that from SOAPdenovo2, the total length of the assembly (273.2 Mb) was ∼100 Mb shorter than expected. Therefore, we used the assembled sequences from SOAPdenovo2 (*k*-mer = 81) in further analyses. Gap sequences of 47.7 million bases, represented by Ns, were filled using the PE reads. After removing sequences from contaminating sources (1.6 Mb from organelles, bacteria, fungi, and humans) and sequences that were shorter than 1,000 bases (97.0 Mb) (see also the next section), the remaining 10,148 sequences were designated PAV_r1.0 (Table 1), which was 272.4 Mb with an N50 length of 219.6 kb (Supplementary Table S3). The GC content was 37.7%, and the length of ambiguous bases (Ns) was 25.6 Mb. The genomic completeness of PAV_r1.0 examined with BUSCO revealed that PAV_r1.0 had 918 (96.0%) complete orthologues and 17 (1.8%) fragmented orthologues, indicating that PAV_r1.0 had good coverage of the gene space of the sweet cherry genome (Supplementary Table S4).

### 3.2. Repetitive sequence analysis

In PAV_r1.0 (273.2 Mb), 119.4 Mb (43.8%) of repetitive sequence was identified (e.g. transposons and retrotransposons), consisting of 34.3 Mb of reported repetitive sequences and 85.1 Mb of repeats unique to PAV_r1.0 (Supplementary Table S5). The reported sequences were predominantly LTR retrotransposons: *Copia* and *Gypsy* elements occupying 8.4 and 8.0 Mb, respectively. On the other hand, repeats occupied 84.2% of the eliminated sequences, each of which was <1,000 bp in length, suggesting that this repeat richness might collapse long assemblies.

### 3.3. Gene predictions and functional annotations

We found 536 tRNA- and 61 rRNA-encoding genes in PAV_r1.0 (Supplementary Tables S6 and S7). Subsequently, we predicted protein-encoding sequences in PAV_r1.0 using evidence-, *ab-initio*-, and homology-based methods in a MAKER pipeline. In the evidence-based method, we used 189,538 transcribed sequences (Supplementary Table S8) obtained from the assembly of 57.6 million transcript reads from eight samples (Supplementary Table S9) to predict 23,709 genes (with .mk suffix), excluding TE-like sequences. Moreover, an additional 19,964 non-TE genes, which did not overlap the 23,709 genes, were predicted using the *ab-initio* method (with .br suffix). In total, 43,349 genes plus 324 pseudogenes were predicted to be in PAV_r1.0 (Supplementary Table S10). The GC content of the coding sequences was 44.3%, and the N50 length was 1,707 bases (Supplementary Table S10).

The 43,349 genes were further annotated using GO, KOG, and KEGG. In the GO analysis, 9,256 (21.4%), 3,610 (8.3%), and 14,582 (33.6%) genes were assigned to the GO slim terms of biological process, cellular component, and molecular function, respectively, (Supplementary Table S11). In the KOG analysis, 2,829, 4,690, and 4,078 genes had significant similarity to genes involved in information storage and processing, cellular processing and signaling, and metabolism (Supplementary Table S12). Furthermore, 1,672 genes were mapped to KEGG metabolic pathways (Supplementary Table S13).

For comparing the genes predicted in PAV_r1.0 with those of other Rosaceae species, the 43,349 genes were clustered with the genes of peach, Japanese apricot, apple, and strawberry to generate 75,627 clusters. A total of 3,459 clusters, including 4,535 genes from sweet cherry, were observed in all investigated species (Supplementary Fig. S3). On the other hand, whereas 869 clusters were absent from only sweet cherry, 16,151 clusters, consisting of 21,642 genes, were specific to sweet cherry. The proportion of *ab initio* genes in the sweet cherry specific clusters, which annotation edit distance (AED)^69^ was 0.28 on average, was 68.4%, while that in other clusters (AED score of 0.16) was 22.3%.

### 3.4. Construction of the consensus genetic map and comparative map

To anchor the genomic sequences to the sweet cherry chromosomes, high-density genetic maps for three F1 populations, C-303, C-309, and HRO, were developed using ddRAD-Seq. Approximately 1.2 million, 1.9 million, and 1.4 million high-quality reads were obtained from ddRAD-Seq libraries for C-303, C-309, and HRO, respectively, and 90.6% of the reads across the three populations aligned to PAV_r1.0; these were used to detect SNP candidates (Supplementary Table S14). After filtering out low-quality candidates, 1,384, 1,475, and 1,157 high-quality SNPs were selected for C-303, C-309, and HRO, respectively. Subsequent linkage analysis, together with 53 and 37 SSRs for C-303 and C-309, respectively, generated eight linkage groups for the parental lines of each population, except for Beniyutaka of C-303 (Supplementary Tables S15 and S16). The six linkage maps were integrated into a consensus linkage map consisting of 2,317 SNPs and 65 SSRs, covering a total of 1,165 cM (Supplementary Fig. S4, Supplementary Tables S15 and S16).

Using the consensus map, the genomic structure of sweet cherry was compared with those of peach (*Pr. persica*), Japanese apricot (*Pr. mume*), strawberry (*F. vesca*), and Chinese pear (*Py. bretschneideri*). Out of the 2,317 mapped SNP loci, the flanking sequences had significant similarity to 2,280 loci in the peach genome, followed by 2,194 in Japanese apricot, 847 in Chinese pear, and 556 in strawberry. The sweet cherry linkage groups were therefore numbered in accordance with the names of peach chromosomes, because there was a one-to-one correspondence between the two genomes (Supplementary Fig. S5).

### 3.5. Establishment of the pseudomolecules

**Figure 1:**
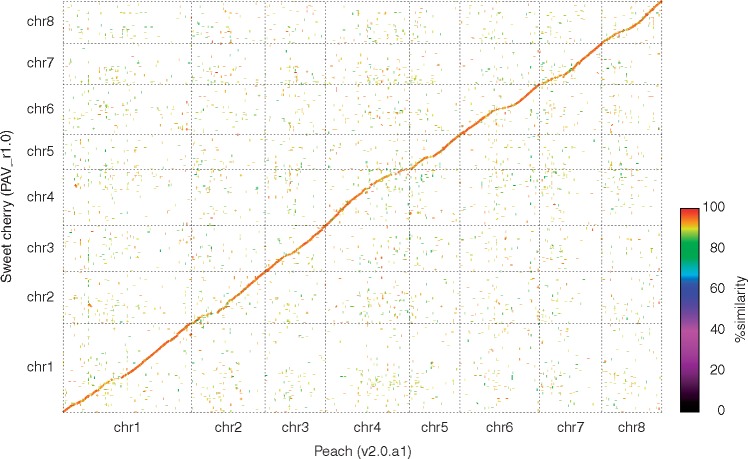
Synteny of the genomes of sweet cherry and peach. X-axis: the genome of peach (GDR v2.0.a1); Y-axis: the genome of sweet cherry (PAV_r1.0). Sequence similarity is indicated by colors.

Pseudomolecules for sweet cherry were established using map- and synteny-based strategies. In the map-based strategy, 162 genomic sequences spanning 14.5 Mb were aligned and ordered on the consensus map using the positions of the 2,280 SNPs as anchors. Furthermore, using the synteny-based strategy, 743 sequences (177.3 Mb) were mapped on the peach genome, with the criterion that peptide sequences of ≥5 continuous genes from a scaffold sequence significantly matched those in the peach genome in the same order. In total, 905 scaffolds, spanning 191.7 Mb (70.4% of the length of PAV_r1.0) and carrying 31,452 genes (72.0% of the predicted genes), were anchored to the sweet cherry chromosomes (Table 2). The scaffold sequences were concatenated with 10,000 Ns into pseudomolecule sequences (Supplementary Table S17). As expected, the pseudomolecules evenly covered 60.2% of the peach genome (Fig. 1).

### 3.6. Genetic diversity analysis

To investigate sequence and structural variation in the sweet cherry genome, whole-genome resequencing was performed on six varieties belonging to a single pedigree (Supplementary Fig. S1). We obtained 28.1× coverage with high-quality sequence read data (9.9 Gb) for each plant line, and 91.6% of the reads were mapped onto the pseudomolecule sequences (Supplementary Table S18).

A total of 1,179,268 sequence variants, consisting of 1,016,866 SNPs and 162,402 insertions/deletions (up to 15 bp differences), were discovered. The densities of SNPs and indels in the genome were estimated to be 412.0 and 65.8 per 100 kb, respectively. Among the SNPs, the major and minor substitutions were G/C to A/T transversions (31.2%) and G/C to C/G transversions (6.0%), respectively, and the transitions/transversions ratio was 1.5 across the six varieties. Differing numbers of sequence variants with respect to PAV_r1.0 were observed, ranging from 527,049 in Benishuho to 640,683 in Benitemari (Supplementary Table S19). The density of the variants in each variety was calculated to be 245.7 variants per 100 kb on average. The number of heterozygous loci was 463,240.5 on average, ranging from 405,911 in Benikirari to 528,752 in Benitemari. Particularly, chromosomes 5 and 7 of Beniyutaka had fewer heterozygous loci.

The SNPs and indels were functionally annotated and classified into four categories: modifiers (88.2%) and moderate- (6.4%), low- (4.6%), and high- (0.7%) impact mutations (Supplementary Table S20). The most prevalent were variants in intergenic regions (modifiers, 65.8%) followed by intron variants (modifiers, 19.2%), missense variants (moderate-impact, 6.1%), and synonymous variants (low-impact, 3.9%). In the high-impact category, frameshift (0.3%) and stop-gained variants (0.2%) dominated.

In addition, CNV candidates were detected over the genomes of the six lines (Supplementary Fig. S6). The average length of CNVs was 2.5 kb and the longest was approximately 32 kb in chromosome 7 of Beniyutaka, which included eight predicted genes (Pav_sc0000496.1_g220.1.br to Pav_sc0000496.1_g310.1.br). Numbers of the CNVs were ranging from 3,341 in Benishuho to 9,074 in Benikirari.

### 3.7. DNA marker development

CAPS and indel markers were developed in accordance with the sequence variants identified from whole-genome resequencing. Out of the 1,016,866 SNPs, 131,679 (12.9%) were located in the recognition sequence of 19 restriction enzymes. We also designed a total of 143,223 CAPS markers (Supplementary Table S21). In parallel, 151,468 indel markers for which primers were available were developed from the 162,402 indels (Supplementary Table S22). A total of 85,731 SSR motifs were detected in PAV_r1.0, including 40,924 (47.7%) di-, 13,473 (15.7%) tri-, 10,340 (12.1%) tetra-, 13,077 (15.3%) penta-, and 7,917 (9.2%) hexa-nucleotide repeat units. The most prevalent sequences in each repeat unit were AG (25,003), AAG (3,919), AAAT (5,551), AAAAT (4,056), and AAAAAT (1,497). We found that 29,539 SSRs (34.5%) were in gene regions and the remaining 56,192 (65.5%) were in intergenic regions. Out of the SSRs, primer pairs were successfully designed for 82,852 SSR motifs (96.6%), which were registered as SSR markers (Supplementary Table S23).

### 3.8. Agronomically important genes in the sweet cherry genome

We compared six agronomically important genes in peach^70^ with the predicted coding sequences. We found that two genes, Pav_sc0000464.1_g250.1.br and Pav_sc0000493.1_g020.1.br, were putative orthologues for ppa016711m (peach skin color) and ppa027093m (peach flesh color), respectively. In addition, Pav_sc0000103.1_g380.1.mk and Pav_sc0001587.1_g070.1.mk were orthologues of ppa010316m and ppa003772m, which associate with fruit hairiness and shape, respectively. Furthermore, Pav_sc0000024.1_g440.1.mk and Pav_sc0000600.1_g890.1.mk might correspond to ppa003772m (fruit adhesion and texture) and ppa006339m (non-acid fruit).

**Figure 2:**
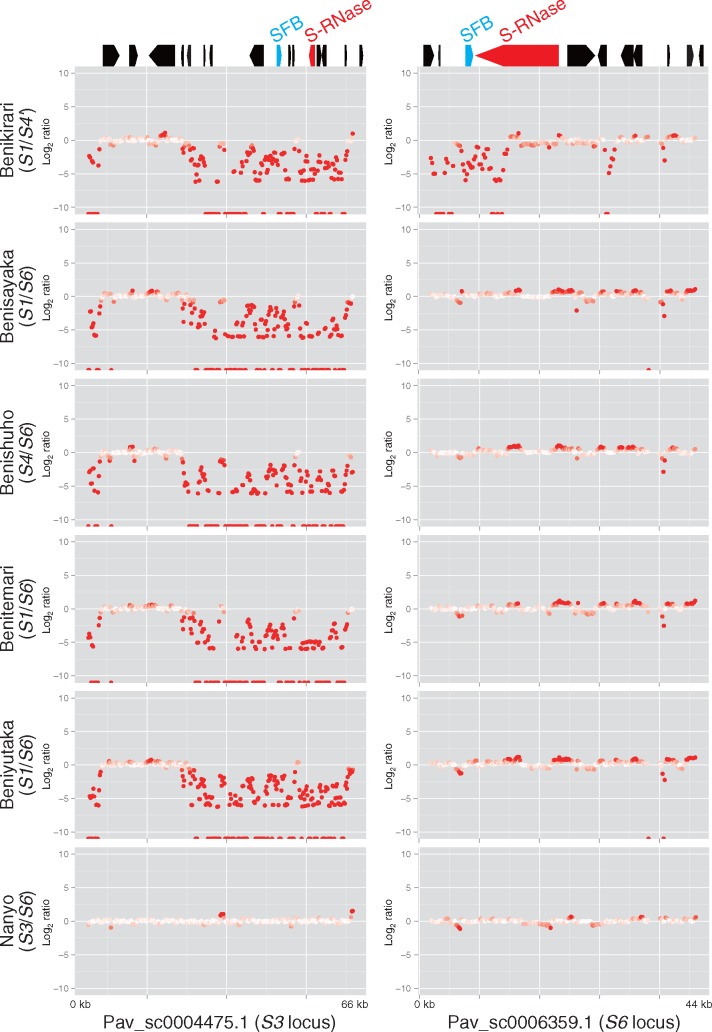
Genomic structures of the *S3* and *S6* loci in Satonishiki and the mapping rate of sequence reads from the six cultivated lines.

Self-incompatibility in sweet cherry is controlled by the *S*-locus, which carries the genes for *S-*RNase and *S*-locus F-box protein (SFB) as female and male determinants, respectively. Satonishiki possesses the *S3* and *S6* haplotypes. As expected, *S*-RNase (Pav_sc0004475.1_g130.1.mk) and *SFB* (Pav_sc0004475.1_g100.1.mk) of the *S3* haplotype were identified in a single contig sequence, Pav_sc0004475.1, and those of *S6* (*S*-RNase: Pav_sc0006359.1_g040.1.mk; and *SFB*: Pav_sc0006359.1_g030.1.mk) were found in another contig, Pav_sc0006359.1. The physical distances between *S*-RNase and *SFB* were 7.4 kb and 1.5 kb in the *S3* and *S6* haplotypes, respectively. Neither the orientation nor the order of the genes predicted in the contigs was conserved, suggesting that the genomic structures of the *S*-loci were divergent. Subsequently, we investigated the depth of coverage of the resequencing across the six lines (Fig. 2). The reads were evenly mapped across the contigs when the lines had an identical *S*-haplotype to Satonishiki. Otherwise, the coverage was partial, suggesting that the genome sequences were divergent across different *S*-haplotypes (e.g. *S1*, *S4*, and *S4’*) as well as *S3* and *S6*.

## 4. Discussion

Here, we report the first draft genome sequence of sweet cherry. The sequence data were used to establish genetic linkage maps with ddRAD-Seq technology, to enable whole-genome resequencing analysis to reveal the genetic diversity of cultivated lines, and to develop genome-wide DNA markers including SNPs, indels, and SSRs. In addition, agronomically important genes were identified by comparative analysis with Rosaceae relatives. This information will further genetic and genomic studies as well as assist in sweet cherry breeding programs.

The size of the assembled genome, PAV_r1.0, was 272.4 Mb, which covered 77.8% of the estimated genome size of ∼350 Mb (Table 1). The remaining 97 Mb of sequence was eliminated from the final assembly due to short contigs (<1,000 bp) enriched in repetitive sequences. The genome sizes of other diploid species in Rosaceae are estimated to be 265 Mb in peach,^2^ 280 Mb in Japanese apricot,^5^ and 240 Mb in strawberry,^6^ all of which are approximately 100 Mb shorter than that of sweet cherry. On the other hand, the proportion of repetitive sequences in PAV_r1.0 was almost equal to that of *Pr. persica*, *Pr. mume*, and *F. vesca*: approximately 40%. Therefore, we considered that a subset of the sweet cherry genome, PAV_r1.0, might correspond to the genomes of *Pr. persica*, *Pr. mume*, and *F. vesca*. Indeed, PAV_r1.0 included >96% of BUSCO genes (Supplementary Table S4), suggesting that PAV_r1.0 sufficiently covered the gene space of the sweet cherry genome. The repeat-rich 97 Mb of sequences eliminated from the assembly might cause genome expansion in sweet cherry, making it larger than the other diploid species.

**Table 1:** Assembly statistics of the sweet cherry genome

	PAV_r1.0
Estimated genome size (bp)	352,883,670
# of scaffolds	10,148
Size of scaffolds (bp)	272,361,615
Scaffold N50 (bp)	219,566
Longest scaffold (bp)	1,460,269
GC (%)	37.7
# of genes	43,673
Mean size of genes (bp)	1,097
Repeat (%)	43.8

**Table 2:** Statistics of the pseudomolecules for the sweet cherry genome

Pseudomolecule	No. of assigned scaffolds	%^a^	Total size of assigned scaffolds (bp)	%^a^	No. of predicted genes	%^a^
PAV_r1.0chr1	161	1.6	41,632,855	15.3	6,737	15.4
PAV_r1.0chr2	111	1.1	24,154,475	8.9	3,949	9.0
PAV_r1.0chr3	86	0.8	21,763,589	8.0	3,588	8.2
PAV_r1.0chr4	128	1.3	26,009,932	9.5	4,126	9.4
PAV_r1.0chr5	57	0.6	16,460,956	6.0	2,822	6.5
PAV_r1.0chr6	159	1.6	23,031,171	8.5	3,838	8.8
PAV_r1.0chr7	85	0.8	19,052,082	7.0	3,180	7.3
PAV_r1.0chr8	118	1.2	19,599,356	7.2	3,212	7.4
Total	905	8.9	191,704,416	70.4	31,452	72.0

^a^Percentage of PAV_r1.0.

Comparative analysis of the consensus genetic map (Supplementary Fig. S4, Supplementary Table S16) found high chromosome-level synteny between sweet cherry and peach (Supplementary Fig. S5), supporting the results of previous studies.^9^ Therefore, it would be possible to apply genetic knowledge from peach to sweet cherry, as proposed by Dirlewanger et al.^10^ As shown above, orthologues of agronomically important genes identified by a genome-wide association study (GWAS) in peach^70^ were nominated in sweet cherry using sequence similarity. In addition, *in silico* mapping of QTLs and genes, which have been curated and summarized in GDR^47^ (http://www.rosaceae.org (8 May 2017, date last accessed)) and PGDBj^71^ (http://pgdbj.jp (8 May 2017, date last accessed)), among other databases, could identify useful gene candidates for sweet cherry breeding programs. Moreover, it might be possible to identify attractive genes from the sweet cherry specific cluster (Supplementary Fig. S3), even though most of genes had no informative functional annotations due to *ab initio* prediction. Whole-genome resequencing is one of the most effective methods for allele mining. In the future, genotype and sequence variation could be assigned to phenotypic variation using QTL studies and GWAS.

In general, whole-genome resequencing of wide-spread cultivated lines and their founders would reveal historical domestication and breeding processes. However, in this study, we targeted six cultivated lines, all of which were bred in Yamagata, Japan, and registered within 40 years. It would be difficult to obtain evidence of historical events, but it is possible to gain insight into new breeding strategies. Because breeding programs for sweet cherry, as well as other fruiting trees, require time and space, it is difficult to perform high-throughput breeding. Therefore, genomics-assisted breeding^72^ and new plant-breeding techniques^73^ are more effective approaches. For example, in pear, genome-wide information about SNPs and phenotypes enables the prediction of trait segregation in a progeny population, which assists in choosing a good parental combination.^74^ Moreover, in apple, accelerating generation advancement has been accomplished through a plant virus vector that carries a promoter for *Arabidopsis FLOWERING LOCUS T* and a silencer for apple *TERMINAL FLOWER 1*.^75^ These technologies developed in Rosaceae, together with a genomic selection strategy,^72^ would make it possible to quickly produce excellent lines, whose phenotypes (e.g. fruit size, taste, and shelf-life) would exceed those of the current leading varieties. The genomic information obtained from this study would accelerate genetic analysis and breeding programs in sweet cherry as well as other fruiting trees.

## 5. Availability

The genome assembly data (scaffold and pseudomolecule sequences), annotations, gene models, genetic maps, and DNA polymorphism are available at DBcherry (http://cherry.kazusa.or.jp/ (8 May 2017, date last accessed)).

## Supplementary Material

Supplementary Data

Supplementary Data

Supplementary Data
