# Supplementary material for: The genome sequence of sweet cherry (Prunus avium) for use in genomics-assisted breeding
Source: DNA Res. 2017 May 25;24(5):499–508. doi: 10.1093/dnares/dsx020 (PMC5737369; doi:10.1093/dnares/dsx020)
Supplement: Supplementary Data [file dsx020_supp_sweetcherrygenome-supplementaryfigures.r1.pdf]

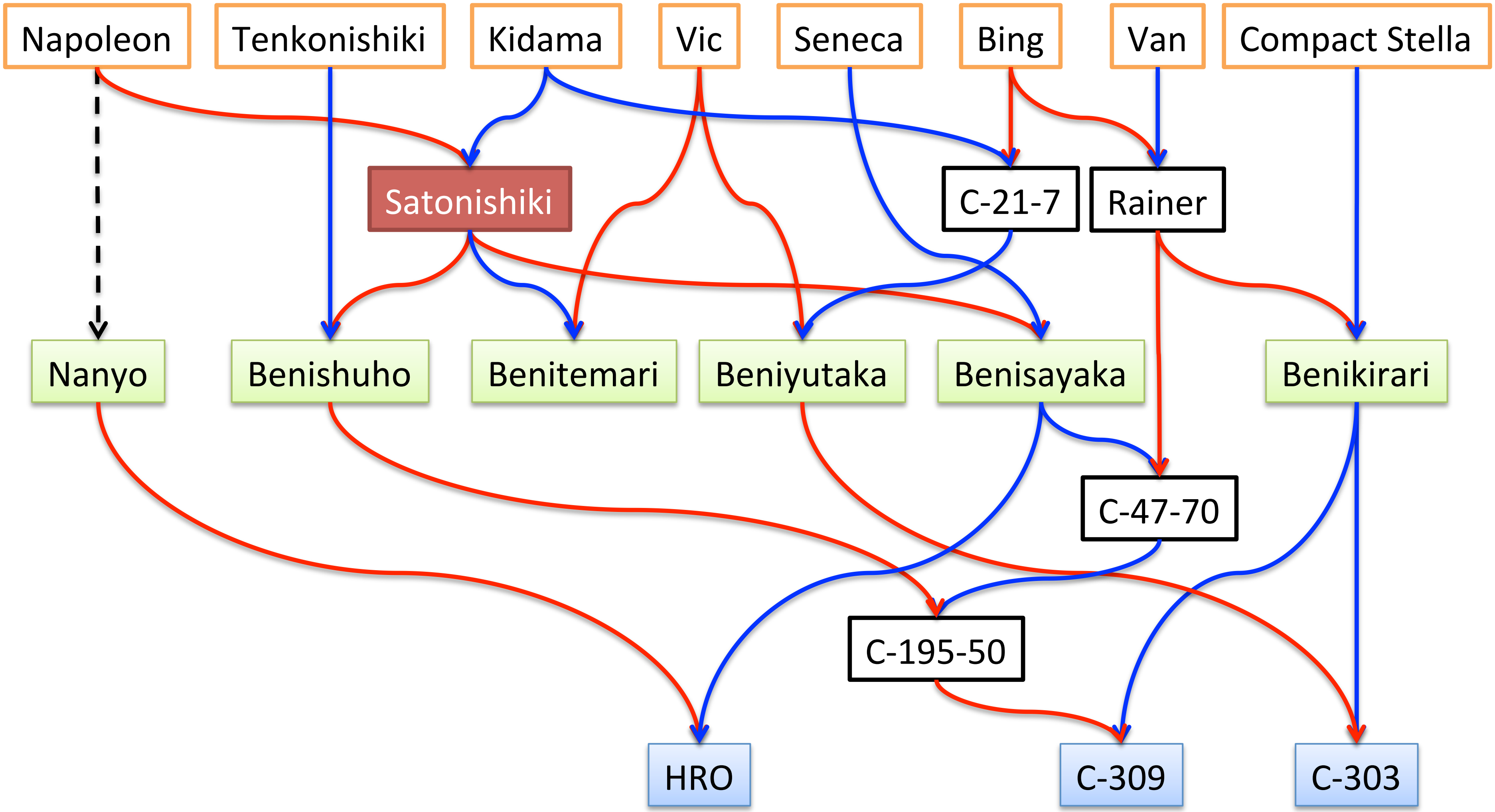

Supplementary Figure S1 Pedigree of the plant materials

Maternal and paternal lines are indicated by red and blue arrows. Nanyo is a progeny of Napoleon, but the paternal line is unknown. The reference line, Satonishiki, is indicated by a red box. Six lines used in whole genome sequencing analysis and eight founders of this pedigree are shown in yellow and orange boxes. HRO, C-309, and C-303 in blues boxes are mapping population. 'Kidama' is a synonym of 'Governor Wood'.

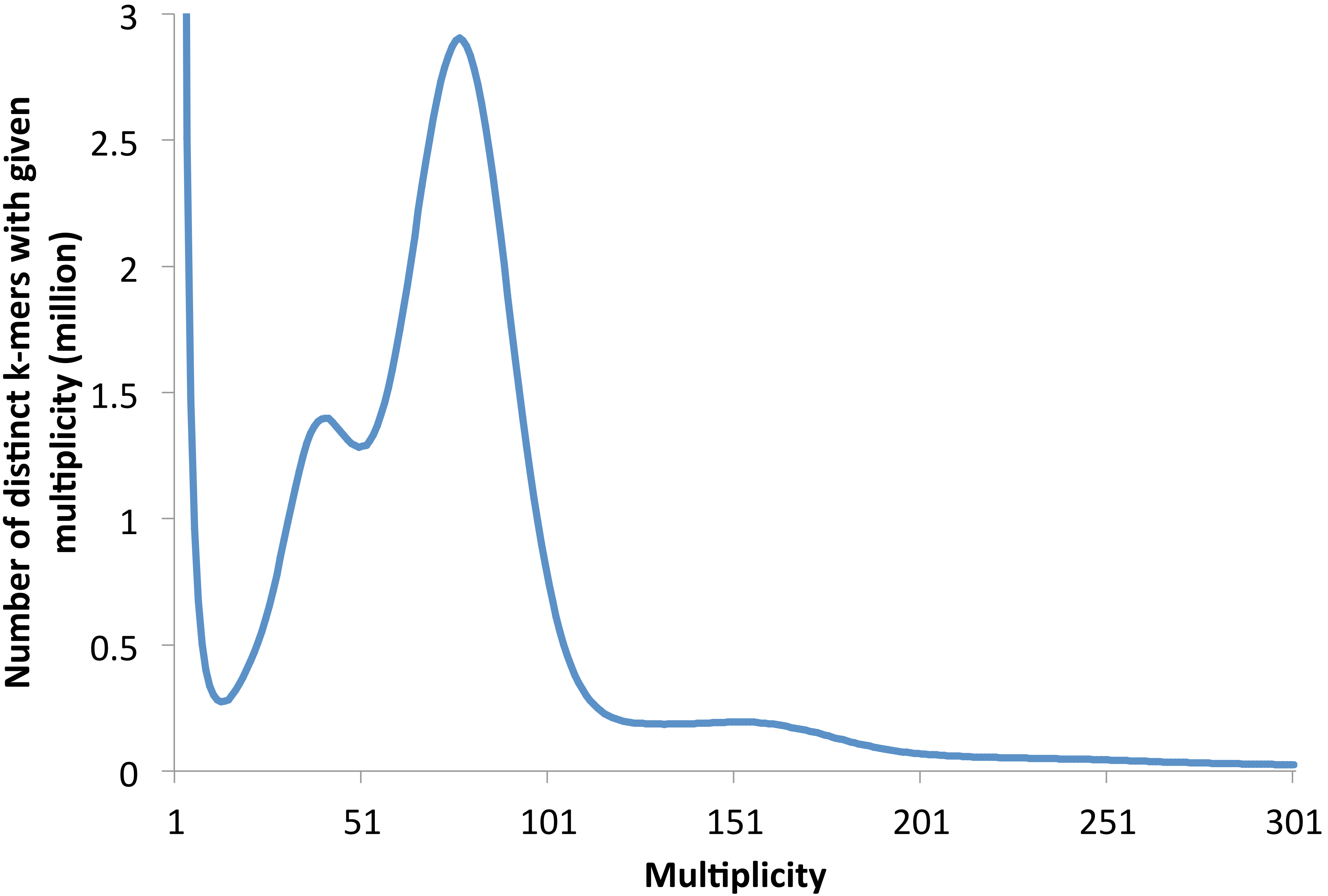

Supplementary Figure S2 Genome size estimations in Satonishiki with the distributions of the number of distinct k-mer (k=17) with the given multiplicity values

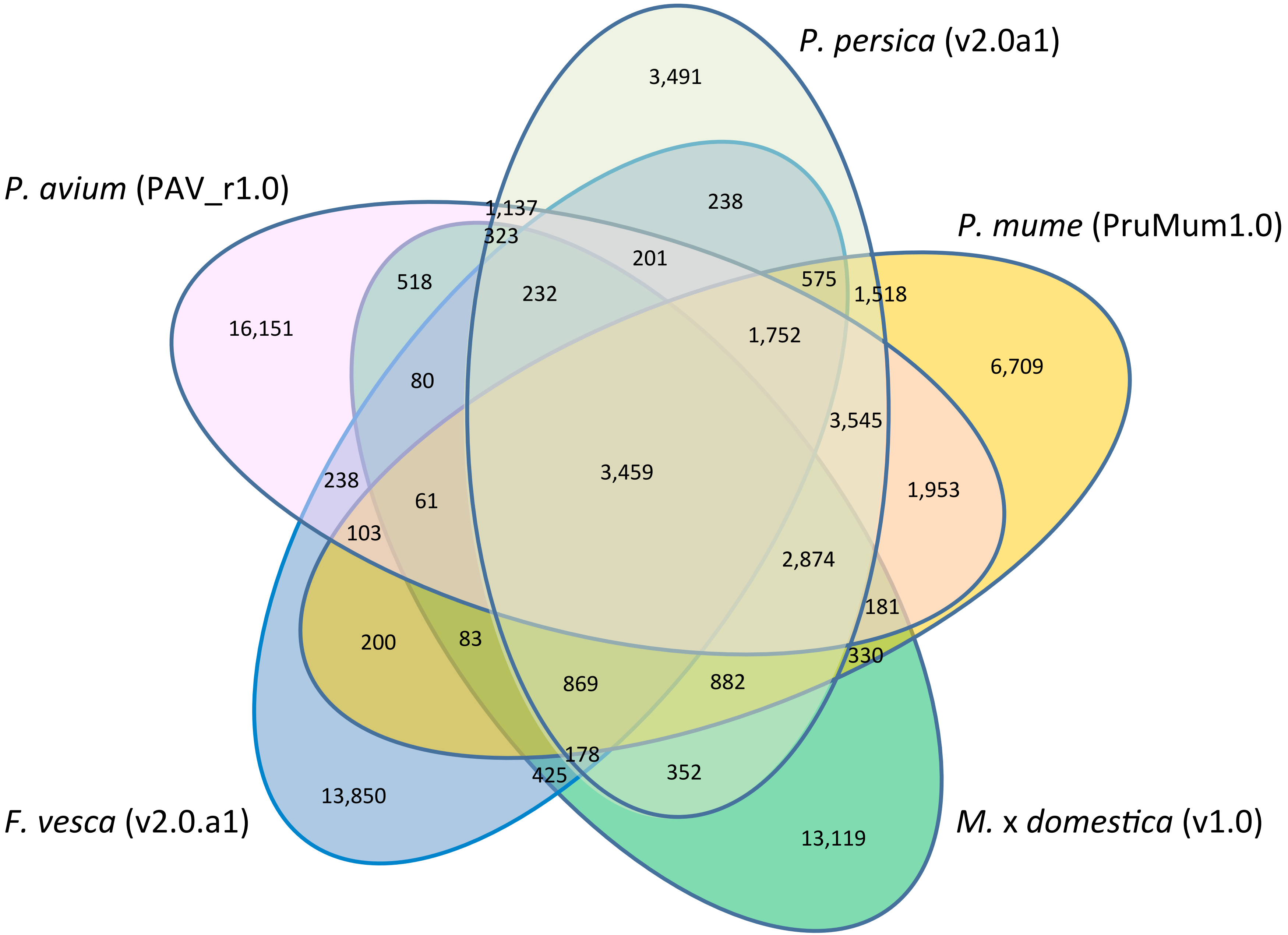

Supplementary Figure S3 Venn diagram showing numbers of gene clusters in sweet cherry and the four Rosaceae species

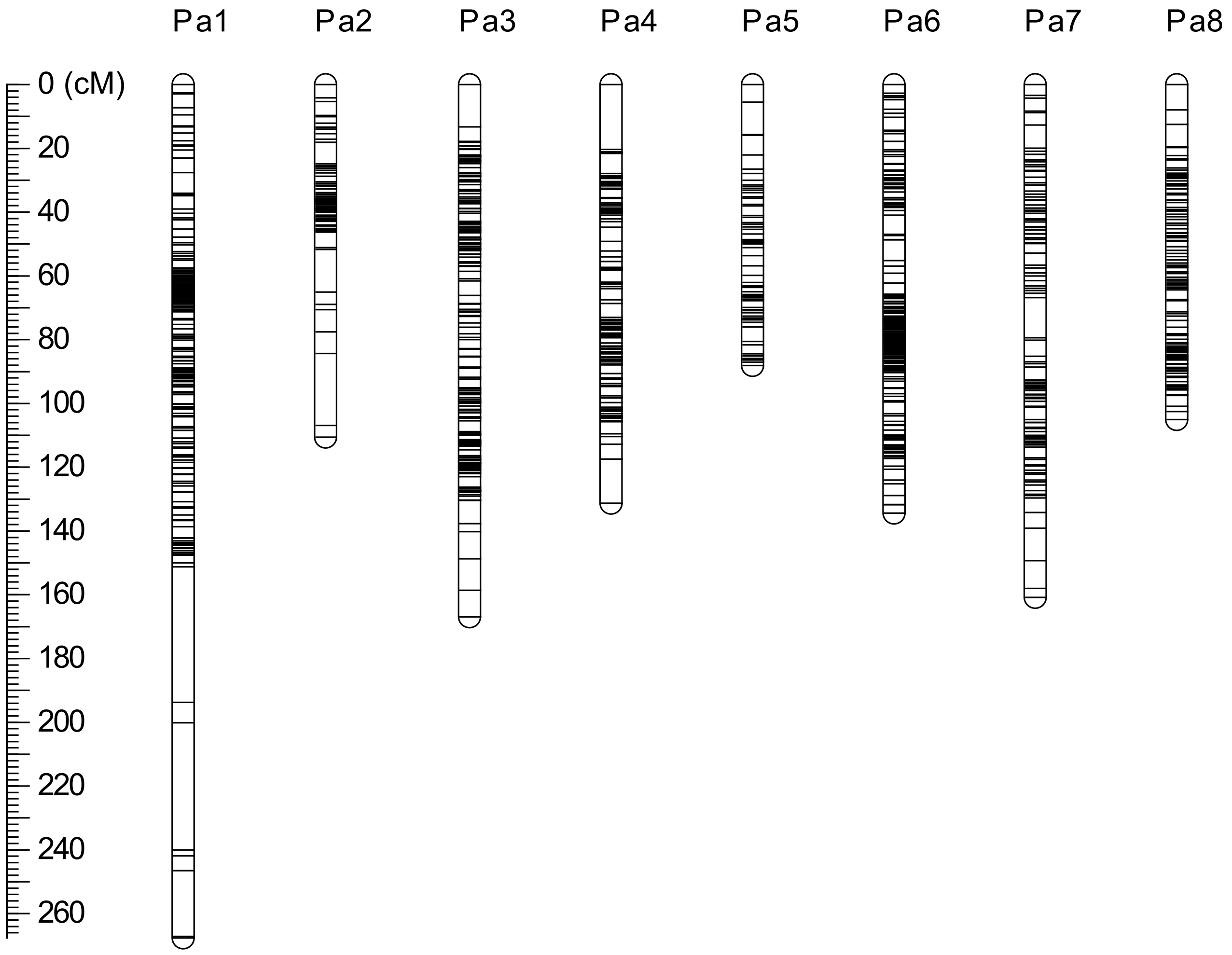

Supplementary Figure S4 An integrated consensus genetic map of sweet cherry

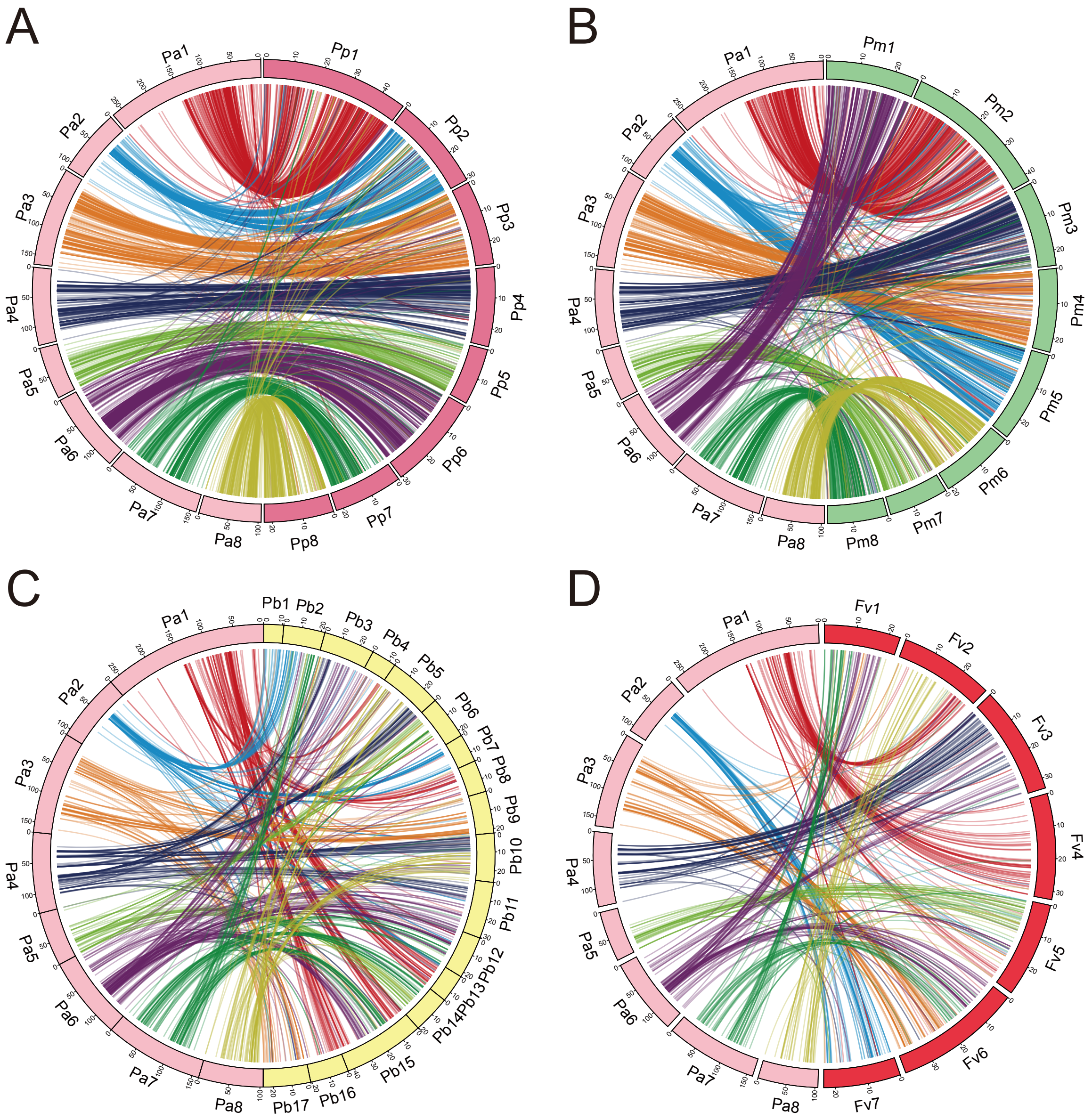

Supplementary Figure S5 Comparative genomics between the genetic map of sweet cherry and the genomes of Rosaceae

Sweet cherry linkage groups represented in map distances (cM) are compared with the genomes (in Mb) of peach (A), Japanese apricot (B), Chinese pear (C), and strawberry (D).

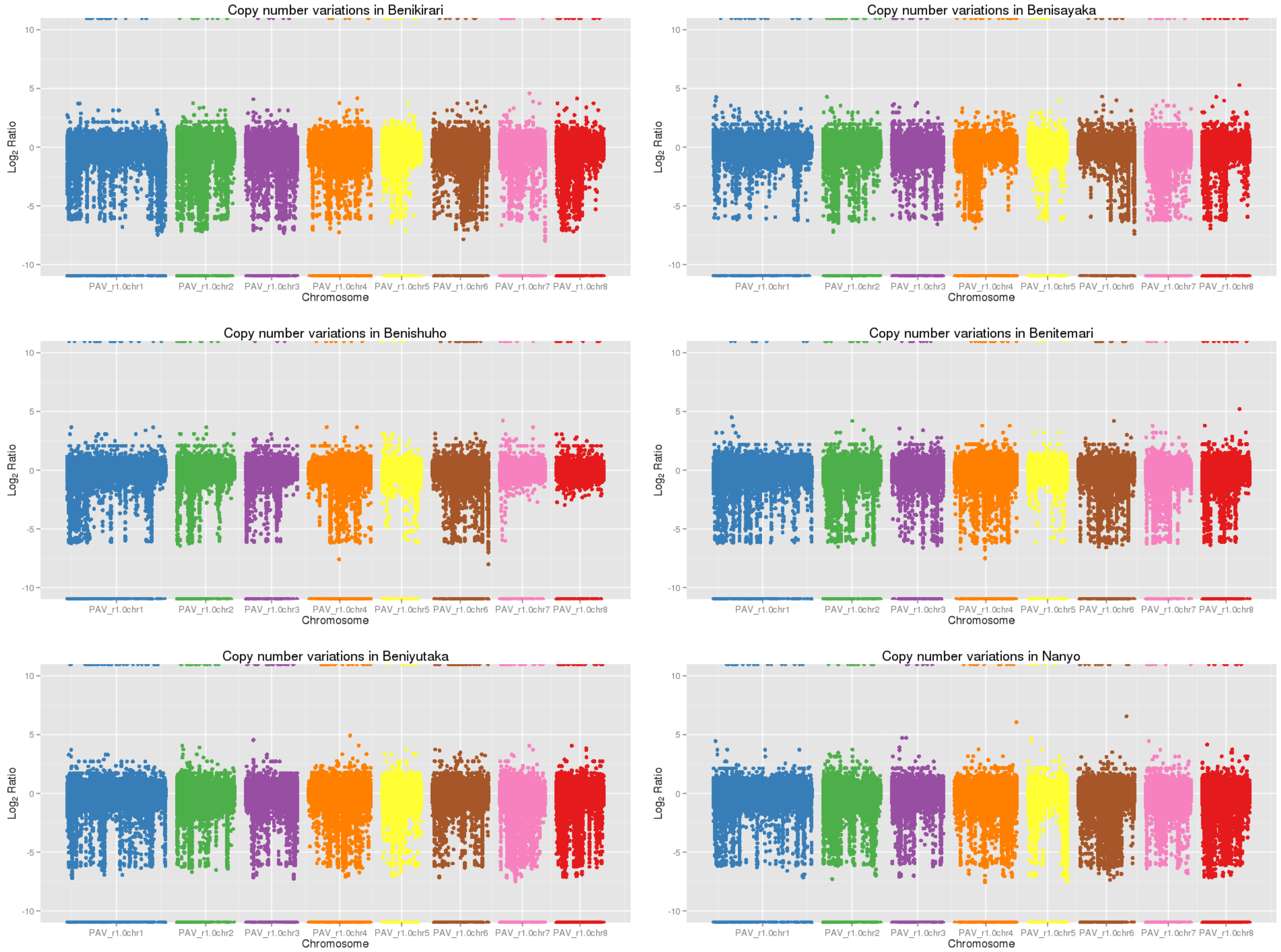

Supplementary Figure S6 Copy number variations in six cultivated sweet cherry cultivars with respect to PAV_r1.0 (Satonishiki)
