# Supplementary material for: The genome sequence of sweet cherry (Prunus avium) for use in genomics-assisted breeding
Source: DNA Res. 2017 May 25;24(5):499–508. doi: 10.1093/dnares/dsx020 (PMC5737369; doi:10.1093/dnares/dsx020)
Supplement: Supplementary Data [file dsx020_supp_sweetcherrygenome-supplementaryinformation.r1.pdf]

Supplementary information

Genomic sequence of sweet cherry (*Prunus avium*) for use in genomics-assisted breeding

Kenta Shirasawa[1*], Kanji Isuzugawa[2], Mitsunobu Ikenaga[3], Yutaro Saito[2], Toshiya Yamamoto[4], Hideki Hirakawa[1], and Sachiko Isobe[1]

[1]Kazusa DNA Research Institute, Kisarazu, Chiba 292-0818, Japan.
[2]Horticultural Experiment Station, Yamagata Integrated Agricultural Research Center, Sagae, Yamagata 991-0043, Japan.
[3]Central Agricultural Experiment Station, Agricultural Research Department, Hokkaido Research Organization, Yubari, Hokkaido 069-1395, Japan.
[4]Institute of Fruit Tree and Tea Science, National Agriculture and Food Research Organization, Japan.

*Corresponding author
Kenta Shirasawa
Kazusa DNA Research Institute
2-6-7 Kazusa-Kamatari, Kisarazu, Chiba 291-0818, Japan
Tel. 81-438 52 3935; fax 81-438 52 3934; email shirasaw@kazusa.or.jp

Supplementary Table S1 SSR markers used for map constructions.
Supplementary Table S2 Sequencing data used for *de novo* genome assembly.
Supplementary Table S3 *De novo* genome assembly statistics.
Supplementary Table S4 Genome assembly completeness examined with BUSCO.
Supplementary Table S5 Length and ratio of repetitive sequences.
Supplementary Table S6 Number of tRNA-encoding genes predicted in PAV_r1.0.
Supplementary Table S7 Number of rRNA-encoding genes predicted in PAV_r1.0.
Supplementary Table S8 RNA-Seq data used for *de novo* transcriptome assembly.
Supplementary Table S9 *De novo* transcriptome assembly statistics.
Supplementary Table S10 Statistics of protein-encoding genes predicted in PAV_r1.0.
Supplementary Table S11 Number of GO terms for the protein-encoding genes.
Supplementary Table S12 Number of KOG functions for the protein-encoding genes.
Supplementary Table S13 Number of genes mapped to KEGG pathways.

Supplementary Table S14 Sequencing data and alignment rate of RAD-Seq for mapping populations.

Supplementary Table S15 Number of marker loci in genetic maps and map lengths.

Supplementary Table S16 Genetic map positions of marker loci.

Supplementary Table S17 Scaffold sequences in the pseudomolecule for the sweet cherry genome.

Supplementary Table S18 Sequencing data and alignment rate of whole-genome resequencing.

Supplementary Table S19 Numbers of SNPs and indels identified from whole-genome resequencing.

Supplementary Table S20 Numbers of annotated SNPs and indels classified by SnpEff program.

Supplementary Table S21 CAPS markers developed from PAV_r1.0.

Supplementary Table S22 Indel markers developed from PAV_r1.0.

Supplementary Table S23 SSR markers developed from PAV_r1.0.

Supplementary Figure S1 Pedigree of the plant materials. Maternal and paternal lines are indicated by red and blue arrows. Nanyo is a progeny of Napoleon, but the paternal line is unknown. The reference line, Satonishiki, is indicated by a red box. The six lines used in whole-genome sequencing and the seven founders of this pedigree are shown in yellow and orange boxes. HRO, C309, and C303, in blue boxes, are the mapping populations.

Supplementary Figure S2 Genome size estimation in Satonishiki, with the distributions of distinct k-mers ($k = 17$) with the given multiplicity values.

Supplementary Figure S3 Venn diagram showing the number of gene clusters in sweet cherry and the four Rosaceae species.

Supplementary Figure S4 An integrated consensus genetic map of sweet cherry.

Supplementary Figure S5 Comparative genomics between the genetic map of sweet cherry and the genomes of peach, Japanese apricot, Chinese pear, and strawberry.

Supplementary Figure S6 Copy number variations in six cultivated sweet cherry cultivars with respect to PAV_r1.0 (Satonishiki).
